# Interaction between vitamin E intake and a *COMT* gene variant on colorectal cancer risk among Korean adults: a case-control study

**DOI:** 10.4178/epih.e2023100

**Published:** 2023-11-14

**Authors:** Shinyoung Jun, Madhawa Gunathilake, Jeonghee Lee, Jae Hwan Oh, Hee Jin Chang, Dae Kyung Sohn, Aesun Shin, Jeongseon Kim

**Affiliations:** 1 Department of Cancer Biomedical Science, National Cancer Center Graduate School of Cancer Science and Policy, Goyang, Korea; 2 Center for Colorectal Cancer, National Cancer Center, Goyang, Korea; 3 Department of Preventive Medicine, Seoul National University College of Medicine, Seoul, Korea; 4 Cancer Research Institute, Seoul National University, Seoul, Korea; 5 Department of Cancer, AI & Digital Health, National Cancer Center Graduate School of Cancer Science and Policy, Goyang, Korea

**Keywords:** Vitamin E, Catechol-O-methyltransferase, Single nucleotide polymorphism, Colorectal cancer, Gene-environment interaction, Case-control studies

## Abstract

**OBJECTIVES:**

Previous human trials have not supported the anticarcinogenic effect of vitamin E despite biological plausibility and considerable epidemiological evidence. A possible explanation for this inconsistency is the interactive effect of the catechol-O-methyltransferase (*COMT*) gene and supplemental vitamin E on cancer. We examined whether a *COMT* gene variant modulates the effect of dietary vitamin E intake on colorectal cancer (CRC) risk.

**METHODS:**

In this case-control study of Korean adults (975 cases and 975 age- and sex-matched controls), dietary vitamin E density (mg/1,000 kcal) was measured using a semiquantitative food frequency questionnaire, *COMT* single nucleotide polymorphism (SNP) rs740603 (A>G) was genotyped, and CRC was verified histologically. We estimated odds ratios (ORs) and 95% confidence intervals (CIs) using unconditional logistic regression models with adjustments for potential confounders.

**RESULTS:**

Higher vitamin E density was associated with a lower risk of CRC (highest vs. lowest quartiles: OR, 0.72; 95% CI, 0.55 to 0.96; p-for-trend=0.002). When stratified by *COMT* SNP rs740603 genotype, the inverse association between vitamin E density and CRC risk was confined to those with at least 1 A allele (≥median vs. <median: OR, 0.63; 95% CI, 0.51 to 0.78). The interaction between rs740603 and vitamin E density was significant (p-for-interaction=0.020). No direct association was observed between *COMT* SNP rs740603 and CRC risk (OR, 1.08; 95% CI, 0.83 to 1.41).

**CONCLUSIONS:**

Our findings support a role for a genetic polymorphism in *COMT* in modifying the association between dietary vitamin E intake and CRC.

## INTRODUCTION

Due to its antioxidant properties, vitamin E has been expected to prevent cancer development [1]. Supporting this hypothesis, a considerable number of epidemiological studies have reported that higher vitamin E intake is associated with reduced risks of various cancer types [2–7]. However, randomized controlled trials that were designed to confirm those epidemiological findings have failed to confirm a role of vitamin E supplementation in cancer prevention [8].

Recent results on gene-environment interactions offered a possible explanation for the inconsistent findings on vitamin E and cancer, suggesting that genetic variants involved in oxidant enzyme activity may have confounded the association between vitamin E and cancer risk [9]. In 2 large randomized controlled trials of vitamin E (alpha-tocopherol) supplementation in populations of European ancestry, polymorphisms in the catechol-O-methyltransferase (*COMT*) gene significantly modified the effect of vitamin E on total cancer incidence [9]. The interactive effects did not reach statistical significance when focusing on specific cancer types, but the pattern of vitamin E being beneficial only among rs4680 Met-allele homozygotes was generally consistent for breast cancer and colorectal cancer (CRC) [9]. Questions for further research included whether the pharmacogenetic effect of vitamin E and *COMT* on cancer prevention extends to dietary vitamin E intake and to specific cancer types.

Therefore, we examined whether a polymorphism in *COMT* modulates the effect of dietary vitamin E intake on CRC risk in a case-control study of Korean adults. CRC is the third most common cancer both in Korea and worldwide, and effective and feasible prevention strategies are necessary [10]. We focused on the single-nucleotide polymorphism (SNP) rs740603 (A>G), which was shown to modify the effect of dietary vitamin E intake on waist circumference in a Spanish case-control study [11].

## MATERIALS AND METHODS

### Study population

CRC case-control data were collected at the National Cancer Center Korea. Case participants were recruited among patients who were newly diagnosed with adenocarcinoma of the colon or rectum based on endoscopic biopsies. There were 2 rounds of data collection: first, from August 2010 to August 2013; and second, from January 2018 to September 2020. Control participants were recruited from cancer-free individuals who came for a health checkup at the National Cancer Center Korea from October 2007 to December 2021 [12].

**Figure 1:**
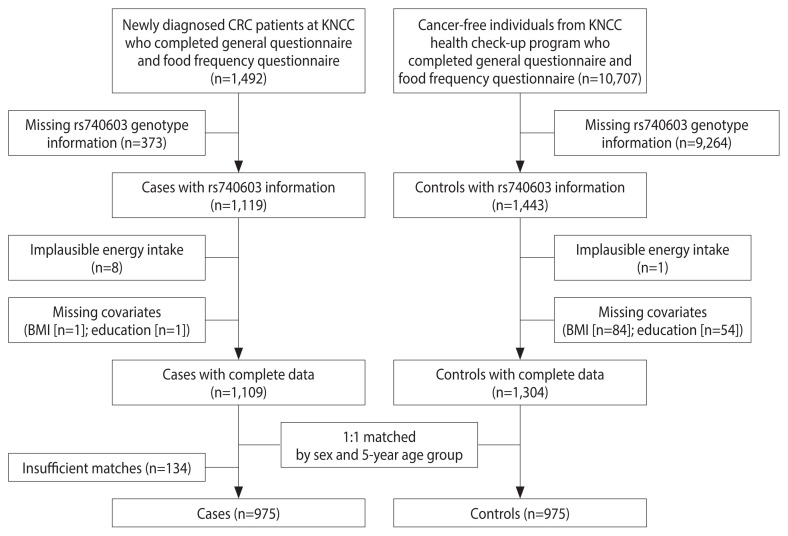
Flowchart of study participant selection. CRC, colorectal cancer; KNCC, National Cancer Center Korea; BMI, body mass index.

The study participant selection process is described in Figure 1. From 1,492 cases and 10,707 controls who completed the general questionnaire and food frequency questionnaire (FFQ), we were able to successfully obtain rs740603 genotype information in 1,119 cases and 1,443 controls. We then excluded those with implausible energy intake (<500 or >4,000 kcal/day regardless of sex [13,14]; 8 cases and 1 control), missing body mass index (BMI) information (1 case and 84 controls), or missing education information (1 case and 54 controls). Among the remaining 1,109 cases and 1,304 controls, controls were matched to cases by sex and 5-year age group at a 1:1 ratio using a published macro [15]. As a result, 975 cases and 975 controls were matched successfully and were included in our analysis.

### Data collection

Trained interviewers administered the general questionnaire on socio-demographic, lifestyle, and health characteristics and the 106-item semiquantitative FFQ. To minimize recall bias, we contacted cases within a few days after the first hospital admission and asked them to report their lifestyles and habitual diets before CRC diagnosis. The FFQ was developed to assess Korean adults’ diets and has been validated against 12-day dietary records across seasons [16]. Daily energy intake and vitamin E intake (i.e., sum of α-, β-, γ-, δ-tocopherols and α-, β-, γ-, δ-tocotrienols) were calculated using the Computer Aided Nutrition Analysis Program version 4 (Can-Pro 4.0; Korean Nutrition Society, Seoul, Korea). To adjust for energy and measurement errors associated with the FFQ [17], vitamin E density was calculated as the ratio of dietary vitamin E intake to total dietary energy intake (mg/1,000 kcal). Vitamin C density was calculated using the same method. We also calculated the Korea Healthy Eating Index, which represents overall dietary quality by measuring adherence to the Dietary Guidelines for Koreans [18].

Anthropometric measurements and a blood draw were conducted by trained health technicians on the same day or within a few days of the administration of the questionnaire. BMI was calculated as weight in kilograms divided by the square of height in meters. Obesity was defined as BMI>25 kg/m^2^ based on the Asian-specific cut-off [19]. If height and body weight measurements were missing (567 cases and 376 controls), self-reported height and weight were used to calculate the BMI. Among 599 participants who had both measured and self-reported anthropometry data, the correlation coefficients between self-reported and measured height and weight were very high (≥0.98).

### Single-nucleotide polymorphism genotyping

From blood samples, DNA was extracted using the MagAttract DNA Blood M48 Kit (Qiagen, Hilden, Germany) and the BioRobot M48 automatic extraction equipment (Qiagen). SNP genotyping was performed using the Illumina MEGA-Expanded Array (Illumina Inc., Hayward, CA, USA) consisting of 123K SNPs. To increase genome coverage, we imputed untyped SNPs, including rs740603, using the Michigan imputation server with the 1000 Genome Project phase 3 East Asian ancestry as a reference panel.

Per *a priori* quality control criteria, we subsequently excluded (1) SNPs with a genotype call rate <98%, (2) individuals with a genotype call rate <98%, (3) SNPs with minor allele frequency (MAF) <5%, (4) SNPs with a Hardy-Weinberg equilibrium p-value <1×10^−6^, and (5) those related based on pairwise identity-by-decent proportion (pi-hat threshold of 0.25).

### Statistical analysis

Descriptive statistics for cases and controls are presented as numbers (%) for categorical variables or means±standard deviations for continuous variables. The significance of differences between cases and controls was determined based on the chi-square test for categorical variables and the t-test for continuous variables.

We used unconditional logistic regression models with adjustment for covariates selected based on the previous literature, biological plausibility, and collinearity to estimate odds ratios (ORs) and 95% confidence intervals (CIs) for the association between vitamin E density or the SNP rs740603 and CRC risk. Vitamin E density was divided into quartiles based on the distribution of controls, and the rs740603 genotype was combined based on a dominant model, with the A allele regarded as a dominant allele because the presence of the A allele was associated with a lower waist circumference and interacted with vitamin E intake [11]. We then examined diet-gene interactions by conducting logistic regression analyses stratified by the rs740603 genotype and assessing the statistical significance of the interaction by using the Wald test for cross-product terms of vitamin E density and the rs740603 genotype. Vitamin E density was divided into 2 groups (< and ≥ median based on the distribution in controls) to secure sufficient statistical power. Model 1 included matching factors (age and sex), total energy intake, and first-degree family history of CRC. Model 2 additionally included socioeconomic and lifestyle factors, such as smoking, drinking, education, and obesity.

In sensitivity analyses, the rs740603 genotype was also tested in both recessive and codominant models (Supplementary Materials 1 and 2). Given the possible synergy between vitamins E and C [20], we also examined joint associations of vitamin E density (< and ≥ median based on the distribution in controls) and vitamin C density (< and ≥ median based on the distribution of controls) with CRC risk stratified by rs740603 genotype (Supplementary Material 3). We also tested the inclusion of the Korean Healthy Eating Index score in addition to all covariates in model 2, but did not present the results due to concerns about overadjustment bias.

All analyses were conducted using SAS version 9.4 (SAS Institute Inc., Cary, NC, USA). All statistical tests were 2-sided and used a significance level of 0.05.

### Ethics statement

This study was approved by the Institutional Review Board of the National Cancer Center Korea (No. NCC 2022-0118), and written informed consent was obtained from all study participants.

## RESULTS

The general characteristics of the case participants and control participants are presented in Table 1. Compared with controls, cases were slightly younger, were less likely to have college degrees and be never-drinkers, and were more likely to have a first-degree family history of CRC. Cases had higher intakes of total energy and vitamin E but had a lower vitamin E density than controls. No significant differences in smoking, obesity, Korean Healthy Eating Index score, or rs740603 genotype between cases and controls were noted.

We first examined the association between vitamin E density and the risk of CRC (Table 2). Statistically significant inverse associations between vitamin E density and CRC risk were observed when adjusted for age, sex, total energy intake, and first-degree family history of CRC without socioeconomic and lifestyle factors (model 1; Q4 vs. Q1: OR, 0.60; 95% CI, 0.46 to 0.78; p-for-trend <0.001) or with socioeconomic and lifestyle factors (model 2; Q4 vs. Q1: OR, 0.72; 95% CI, 0.55 to 0.96; p-for-trend=0.002).

We also examined the relationship between *COMT* SNP rs740603 (G/G vs. A/A or A/G; dominant model) and the risk of CRC (Table 3). No significant association was observed (model 2; OR, 1.08; 95% CI, 0.83 to 1.41). No significant association was noted in either the recessive or codominant model (Supplementary Material 1).

We then examined whether the association between vitamin E density and CRC risk varied according to the SNP rs740603 genotype (Table 4). The inverse association was confined to those with at least one A allele (model 2; p-for-interaction=0.02). Compared with lower vitamin E density (<median), higher vitamin E density (≥median) was associated with an OR of 0.63 (model 2; 95% CI, 0.51 to 0.78) among those with at least 1 A allele (A/A or A/G). Vitamin E density was not associated with CRC risk among those with the G/G genotype (model 2; OR, 1.13; 95% CI, 0.66 to 1.91). The results were consistent in the codominant model, with significant associations between vitamin E density and CRC risk only noted in those with A/A and A/G genotypes (Supplementary Material 2). In the recessive model, significant associations between vitamin E density and CRC risk were observed not only in those with the A/A genotype but also in those with the G/G or A/G genotype, which may have been driven by a significant association among those with the A/G genotype.

In the joint analysis of vitamin C and vitamin E density, vitamin E largely drove the associations. Briefly, significant associations were observed only between the higher dietary vitamin E density group, with either lower or higher vitamin C density, and CRC risk among participants with at least 1 A allele (Supplementary Material 3).

## DISCUSSION

Despite biological plausibility [1] and considerable epidemiological evidence [6,21–23] suggesting that vitamin E plays a protective role in CRC development [8], human trials have not supported the anticarcinogenic effect of supplemental vitamin E. A possible explanation for this inconsistency is the interactive effect of *COMT* and supplemental vitamin E on cancer [9]. Our results suggest that the interaction observed among participants of European ancestry [9] may be applicable to dietary vitamin E and CRC among participants of Asian ethnicity. In our study of Korean adults, dietary vitamin E intake was protective against CRC development only in carriers of the *COMT* rs740603 A allele, but not in those who were homozygotes for the G allele.

The enzyme encoded by *COMT* catalyzes the O-methylation of various compounds, including catecholamines, catechol estrogens, and dietary polyphenols. Therefore, the *COMT* enzyme has been implicated in multiple neuropsychiatric disorders [24,25] and metabolic disorders [11,26–28]. However, insignificant associations have been reported between *COMT* and CRC incidence and survival [29,30], suggesting that *COMT* might interact with environmental factors in modulating carcinogenesis. Indeed, in our study, *COMT* rs740603 was not directly associated with CRC, but rather modified the association between dietary vitamin E and CRC risk. The SNP rs740603 is located within intron 1 and is approximately 6.1 kb 5’ to rs4680, the most studied functional variant. This polymorphism was identified as a potentially functional variant associated with a psychiatric phenotype [31] and had interactive effects with dietary vitamin E intake on waist circumference [11]. We were unable to examine whether rs4680 has similar effects to those of rs740603 due to a lack of genotype data, but a weak correlation has been found between rs4680 and rs740603 in East Asian populations (1000 Genomes; R^2^=0.11) [32]. Future studies could investigate whether rs4680 or other variants correlated with rs740603 may be involved in the interaction between rs740603 and dietary vitamin E on CRC.

The mechanisms underlying this interaction between vitamin E and *COMT* need further investigation, but the interaction may be related to oxidative stress management based on the following data. The knockdown of *COMT* expression was shown to increase estradiol-induced or catechol estrogen-induced reactive oxygen species, microsatellite instability, and neoplastic transformation of endometrial glandular cells [33]. In platelets, the *COMT* enzyme was shown to methylate quercetin, which is an antioxidant [34]. A recent report on the interactive effect of polymorphisms of *IL-10* and *COMT* may have implications regarding the potential interplay between the dopaminergic system and the inflammatory system [35]. However, the possible role of vitamin C in this interaction proposed by a previous study [9] based on its association with the *COMT* gene [36], as well as its synergistic interaction with vitamin E [20], was not supported in our study, suggesting that vitamin E may be involved in a unique mechanism.

In the absence of stratification by rs740603, we observed that dietary vitamin E intake was inversely associated with CRC risk, which is consistent with many previous epidemiological studies [6,21–23]. Because the G allele is a minor allele in the Asian population (MAF of 0.41 in this study sample and 0.40 in the East Asian population from 1000 Genomes), null associations in G allele homozygotes seem diluted when all rs740603 genotypes are combined. Regarding the null findings from previous human trials [8] and some epidemiological studies [37,38], we speculate that the composition (only alpha-tocopherol) and high dose of supplemental vitamin E provided in the trial [1] or the large variation in linkage disequilibrium patterns across populations (especially between European and East Asian populations) [39] may have affected the results.

Our data should be understood within the context of the study’s strengths and limitations. The strengths of our study include histological verification of CRC patients and the use of a validated FFQ. In addition, we adjusted for a range of plausible confounders measured by a comprehensive questionnaire and clinical assessments, although there is always a possibility of residual confounding.

One of the major limitations of this study was the absence of other populations to test the replicability of our results. Moreover, the case-control study design employed in our study is susceptible to recall bias and selection bias. To minimize recall bias, we interviewed CRC patients immediately after hospital admission for surgery and asked them to report their habitual diet and lifestyle prior to diagnosis. To minimize selection bias, we selected control participants from those who visited the same hospital for health check-ups supported by the Korea National Screening Program; however, the control participants who voluntarily came for check-ups may have been more health-conscious than the case participants. Another major limitation is the lack of supplemental vitamin E intake information. As dietary supplements are used by approximately 25% of Korean adults [40], we suggest that future investigations should be conducted with complete information on dietary and supplemental vitamin E information to confirm our findings. Of note, our vitamin E estimate was a simple sum of four tocopherols (α, β, γ, δ) and four tocotrienols (α, β, γ, δ) in milligrams per day. Future studies may also explore the different chemical forms of vitamin E given the growing attention on the anticarcinogenic effects of γ- and δ-tocopherols [41].

In conclusion, the *COMT* genetic variant modified the association between dietary vitamin E intake and CRC; specifically, vitamin E was inversely associated with CRC risk only in carriers of the *COMT* rs740603 A allele. These findings add key evidence to the literature on nutrient-gene interactions affecting CRC risk and may inform future precision nutrition research and practice in cancer prevention.

## Figures and Tables

**Table 1.** Characteristics of colorectal cancer cases and controls

Characteristics	Cases (n=975)	Controls (n=975)	p-value^1^
Age (yr)	56.3±8.9	57.2±8.8	0.015
Sex			>0.999
Male	605 (62.1)	605 (62.1)	
Female	370 (38.0)	370 (38.0)	
Education			<0.001
Less than high school	300 (30.8)	123 (12.6)	
High school graduate or associate’s degree	411 (42.2)	415 (42.6)	
College graduate or more	264 (27.1)	437 (44.8)	
Smoking			0.368
Current	481 (49.3)	457 (46.9)	
Past	344 (35.3)	347 (35.6)	
Never	150 (15.4)	171 (17.5)	
Drinking			
Current	370 (38.0)	295 (30.3)	<0.001
Past	146 (15.0)	94 (9.6)	
Never	459 (47.1)	586 (60.1)	
Obesity (BMI>25 kg/m^2^)	352 (36.1)	339 (34.8)	0.538
First-degree family history of colorectal cancer	91 (9.3)	62 (6.4)	0.015
Total energy intake (kcal/day)	1,938.0±558.7	1,648.0±564.1	<0.001
Vitamin E intake (mg/day)	9.6±4.1	8.6±4.1	<0.001
Vitamin E density (mg/1,000 kcal)	4.9±1.3	5.2±1.5	<0.001
Korean Healthy Eating Index	58.1±9.2	58.8±10.8	0.150
rs740603 genotype			0.998
A/A	336 (34.5)	336 (34.5)	
A/G	483 (49.5)	482 (49.4)	
G/G	156 (16.0)	157 (16.1)	

Values are presented as means±standard deviation or number (%). BMI, body mass index.

1 Differences between cases and controls were tested using the chi-square test for categorical variables and the t-test for continuous variables.

**Table 2.** Association between vitamin E density and colorectal cancer risk

Vitamin E density (mg/1,000 kcal)	No. of cases/controls	Model 1^1^	Model 2^2^
Q1 (≤4.16)	308/243	1.00 (reference)	1.00 (reference)
Q2 (>4.16 and ≤5.03)	279/244	0.78 (0.61, 0.999)	0.84 (0.64, 1.09)
Q3 (>5.03 and ≤5.93)	176/244	0.47 (0.36, 0.62)	0.55 (0.41, 0.73)
Q4 (>5.93)	212/244	0.60 (0.46, 0.78)	0.72 (0.55, 0.96)
p-for-linear trend		<0.001	0.002

Values are presented as odds ratio (95% confidence interval).

1 Adjusted for age, sex, total energy intake and first-degree family history of colorectal cancer.

2 Adjusted for age, sex, total energy intake, first-degree family history of colorectal cancer, smoking, drinking, education, and obesity.

**Table 3.** Association between the rs740603 genotype and CRC risk

*COMT* SNP rs740603^1^	No. of cases/controls	Model 1^2^	Model 2^3^
G/G	156/157	1.00 (reference)	1.00 (reference)
A/A+A/G	819/818	1.07 (0.84, 1.38)	1.08 (0.83, 1.41)

Values are presented as odds ratio (95% confidence interval). CRC, colorectal cancer; COMT, catechol-O-methyltransferase; SNP, single-nucleotide polymorphism.

1 Dominant model.

2 Adjusted for age, sex, total energy intake and first-degree family history of CRC.

3 Adjusted for age, sex, total energy intake, first-degree family history of CRC, smoking, drinking, education, and obesity.

**Table 4.** Association between vitamin E density and CRC risk, stratified by *COMT* rs740603 genotype

*COMT* SNP rs740603^1^	Lower vitamin E^2^	Higher vitamin E^2^	p-value
G/G			
No. of cases/controls	89/89	67/68	
Model 1^3^	1.00 (reference)	0.88 (0.54, 1.43)	0.611
Model 2^4^	1.00 (reference)	1.13 (0.66, 1.91)	0.662
A/A+A/G			
No. of cases/controls	498/398	321/420	
Model 1^3^	1.00 (reference)	0.56 (0.46, 0.69)	<0.001
Model 2^4^	1.00 (reference)	0.63 (0.51, 0.78)	<0.001

Values are presented as odds ratio (95% confidence interval). CRC, colorectal cancer; COMT, catechol-O-methyltransferase; SNP, single-nucleotide polymorphism.

1 Dominant model.

2 Lower and higher vitamin E groups had vitamin E density at or below and above the median (5.03 mg/1,000 kcal), respectively.

3 Adjusted for age, sex, total energy intake and first-degree family history of CRC; p-for-interaction=0.060.

4 Adjusted for age, sex, total energy intake, first-degree family history of CRC, smoking, drinking, education, and obesity; p-for-interaction=0.020.

## Data Availability

The datasets used and analyzed in the current study are available from the corresponding author on reasonable request.

## References

[1] Jiang Q (2019). Natural forms of vitamin E and metabolites-regulation of cancer cell death and underlying mechanisms. IUBMB Life.

[2] Stolzenberg-Solomon RZ, Sheffler-Collins S, Weinstein S, Garabrant DH, Mannisto S, Taylor P (2009). Vitamin E intake, alpha-tocopherol status, and pancreatic cancer in a cohort of male smokers. Am J Clin Nutr.

[3] Huang J, Weinstein SJ, Yu K, Männistö S, Albanes D (2020). A prospective study of serum vitamin E and 28-year risk of lung cancer. J Natl Cancer Inst.

[4] Lawrence WR, Lim JE, Huang J, Weinstein SJ, Männistö S, Albanes D (2022). A 28-year prospective analysis of serum vitamin E, vitamin E-related genetic variation and risk of prostate cancer. Prostate Cancer Prostatic Dis.

[5] Nagel G, Linseisen J, van Gils CH, Peeters PH, Boutron-Ruault MC, Clavel-Chapelon F (2010). Dietary beta-carotene, vitamin C and E intake and breast cancer risk in the European Prospective Investigation into Cancer and Nutrition (EPIC). Breast Cancer Res Treat.

[6] Park Y, Spiegelman D, Hunter DJ, Albanes D, Bergkvist L, Buring JE (2010). Intakes of vitamins A, C, and E and use of multiple vitamin supplements and risk of colon cancer: a pooled analysis of prospective cohort studies. Cancer Causes Control.

[7] Egnell M, Fassier P, Lécuyer L, Gonzalez R, Zelek L, Vasson MP (2017). Antioxidant intake from diet and supplements and risk of digestive cancers in middle-aged adults: results from the prospective NutriNet-Santé cohort. Br J Nutr.

[8] O’Connor EA, Evans CV, Ivlev I, Rushkin MC, Thomas RG, Martin A (2022). Vitamin and mineral supplements for the primary prevention of cardiovascular disease and cancer: updated evidence report and systematic review for the US Preventive Services Task Force. JAMA.

[9] Hall KT, Buring JE, Mukamal KJ, Vinayaga Moorthy M, Wayne PM, Kaptchuk TJ (2019). COMT and alpha-tocopherol effects in cancer prevention: gene-supplement interactions in two randomized clinical trials. J Natl Cancer Inst.

[10] Kang MJ, Won YJ, Lee JJ, Jung KW, Kim HJ, Kong HJ (2022). Cancer statistics in Korea: incidence, mortality, survival, and prevalence in 2019. Cancer Res Treat.

[11] Mansego ML, De Marco G, Ivorra C, Lopez-Izquierdo R, Morcillo S, Rojo-Martínez G (2015). The nutrigenetic influence of the interaction between dietary vitamin E and TXN and COMT gene polymorphisms on waist circumference: a case control study. J Transl Med.

[12] Kim J (2014). Cancer screenee cohort study of the National Cancer Center in South Korea. Epidemiol Health.

[13] Willett W (2013). Nutritional epidemiology.

[14] Santos DA, Silva AM, Matias CN, Magalhães JP, Fields DA, Minderico CS (2014). Validity of a combined heart rate and motion sensor for the measurement of free-living energy expenditure in very active individuals. J Sci Med Sport.

[15] Mortensen LQ, Andresen K, Burcharth J, Pommergaard HC, Rosenberg J (2019). Matching cases and controls using SAS^®^ software. Font Big Data.

[16] Ahn Y, Kwon E, Shim JE, Park MK, Joo Y, Kimm K (2007). Validation and reproducibility of food frequency questionnaire for Korean genome epidemiologic study. Eur J Clin Nutr.

[17] Freedman LS, Commins JM, Moler JE, Willett W, Tinker LF, Subar AF (2015). Pooled results from 5 validation studies of dietary self-report instruments using recovery biomarkers for potassium and sodium intake. Am J Epidemiol.

[18] Yun S, Park S, Yook SM, Kim K, Shim JE, Hwang JY (2022). Development of the Korean Healthy Eating Index for adults, based on the Korea National Health and Nutrition Examination Survey. Nutr Res Pract.

[19] Seo MH, Lee WY, Kim SS, Kang JH, Kang JH, Kim KK (2019). 2018 Korean Society for the Study of Obesity guideline for the management of obesity in Korea. J Obes Metab Syndr.

[20] Niki E (1987). Interaction of ascorbate and alpha-tocopherol. Ann N Y Acad Sci.

[21] Leenders M, Leufkens AM, Siersema PD, van Duijnhoven FJ, Vrieling A, Hulshof PJ (2014). Plasma and dietary carotenoids and vitamins A, C and E and risk of colon and rectal cancer in the European Prospective Investigation into Cancer and Nutrition. Int J Cancer.

[22] Luo H, Fang YJ, Lu MS, Pan ZZ, Huang J, Chen YM (2019). Dietary and serum vitamins A and E and colorectal cancer risk in Chinese population: a case-control study. Eur J Cancer Prev.

[23] Alves Ribeiro RR, Rolim de Brito I, Andrade Souza K, de Castro Souza L, Almeida de Oliveira T, Weller M (2022). Risk of colorectal cancer in a Brazilian population is differentially associated with the intake of processed meat and vitamin E. Nutr Cancer.

[24] Pap D, Gonda X, Molnar E, Lazary J, Benko A, Downey D (2012). Genetic variants in the catechol-o-methyltransferase gene are associated with impulsivity and executive function: relevance for major depression. Am J Med Genet B Neuropsychiatr Genet.

[25] Fiocco AJ, Lindquist K, Ferrell R, Li R, Simonsick EM, Nalls M (2010). COMT genotype and cognitive function: an 8-year longitudinal study in white and black elders. Neurology.

[26] Htun NC, Miyaki K, Song Y, Ikeda S, Shimbo T, Muramatsu M (2011). Association of the catechol-O-methyl transferase gene Val158Met polymorphism with blood pressure and prevalence of hypertension: interaction with dietary energy intake. Am J Hypertens.

[27] Voutilainen S, Tuomainen TP, Korhonen M, Mursu J, Virtanen JK, Happonen P (2007). Functional COMT Val158Met polymorphism, risk of acute coronary events and serum homocysteine: the Kuopio ischaemic heart disease risk factor study. PLoS One.

[28] Hall KT, Nelson CP, Davis RB, Buring JE, Kirsch I, Mittleman MA (2014). Polymorphisms in catechol-O-methyltransferase modify treatment effects of aspirin on risk of cardiovascular disease. Arterioscler Thromb Vasc Biol.

[29] Landi S, Gemignani F, Moreno V, Gioia-Patricola L, Chabrier A, Guino E (2005). A comprehensive analysis of phase I and phase II metabolism gene polymorphisms and risk of colorectal cancer. Pharmacogenet Genomics.

[30] Passarelli MN, Newcomb PA, Makar KW, Burnett-Hartman AN, Phipps AI, David SP (2014). No association between germline variation in catechol-O-methyltransferase and colorectal cancer survival in postmenopausal women. Menopause.

[31] Ittiwut R, Listman JB, Ittiwut C, Cubells JF, Weiss RD, Brady K (2011). Association between polymorphisms in catechol-O-methyltransferase (COMT) and cocaine-induced paranoia in European-American and African-American populations. Am J Med Genet B Neuropsychiatr Genet.

[32] Alexander TA, Machiela MJ (2020). LDpop: an interactive online tool to calculate and visualize geographic LD patterns. BMC Bioinformatics.

[33] Salama SA, Kamel M, Awad M, Nasser AH, Al-Hendy A, Botting S (2008). Catecholestrogens induce oxidative stress and malignant transformation in human endometrial glandular cells: protective effect of catechol-O-methyltransferase. Int J Cancer.

[34] Wright B, Gibson T, Spencer J, Lovegrove JA, Gibbins JM (2010). Platelet-mediated metabolism of the common dietary flavonoid, quercetin. PLoS One.

[35] Wang J, Xu H, Wang D, Wei G, Zhou H, Wang L (2021). The interactive effect of genetic polymorphisms of IL-10 and COMT on cognitive function in schizophrenia. J Psychiatr Res.

[36] Shin SY, Fauman EB, Petersen AK, Krumsiek J, Santos R, Huang J (2014). An atlas of genetic influences on human blood metabolites. Nat Genet.

[37] Wang Z, Joshi AM, Ohnaka K, Morita M, Toyomura K, Kono S (2012). Dietary intakes of retinol, carotenes, vitamin C, and vitamin E and colorectal cancer risk: the Fukuoka colorectal cancer study. Nutr Cancer.

[38] Heine-Bröring RC, Winkels RM, Renkema JM, Kragt L, van Orten-Luiten AC, Tigchelaar EF (2015). Dietary supplement use and colorectal cancer risk: a systematic review and meta-analyses of prospective cohort studies. Int J Cancer.

[39] Mukherjee N, Kidd KK, Pakstis AJ, Speed WC, Li H, Tarnok Z (2010). The complex global pattern of genetic variation and linkage disequilibrium at catechol-O-methyltransferase. Mol Psychiatry.

[40] Kim S, Song Y, Lee JE, Jun S, Shin S, Wie GA (2017). Total antioxidant capacity from dietary supplement decreases the likelihood of having metabolic syndrome in Korean adults. Nutrients.

[41] Yang CS, Luo P, Zeng Z, Wang H, Malafa M, Suh N (2020). Vitamin E and cancer prevention: studies with different forms of tocopherols and tocotrienols. Mol Carcinog.

