# Supplementary material for: Interaction between vitamin E intake and a COMT gene variant on colorectal cancer risk among Korean adults: a case-control study
Source: Epidemiol Health. 2023 Nov 14;45:e2023100. doi: 10.4178/epih.e2023100 (PMC10876447; doi:10.4178/epih.e2023100)
Supplement: Supplementary file 1 [file epih-45-e2023100-Supplementary-1.docx]

| **Supplemental Material 1. Association between rs740603 genotype (recessive or codominant model) and colorectal cancer risk** | | | |
| --- | --- | --- | --- |
| ***COMT* SNP rs740603** | **Number of cases/controls** | **Model 1**  **OR (95% CI)^1^** | **Model 2**  **OR (95% CI)^2^** |
| *Recessive* |  |  |  |
| G/G+A/G | 639/639 | 1 (ref) | 1 (ref) |
| A/A | 336/336 | 1.01 (0.83, 1.22) | 0.995 (0.81, 1.22) |
| *Codominant* |  |  |  |
| G/G | 156/157 | 1 (ref) | 1 (ref) |
| A/G | 483/482 | 1.08 (0.83, 1.40) | 1.10 (0.83, 1.45) |
| A/A | 336/336 | 1.07 (0.81, 1.41) | 1.07 (0.80, 1.43) |

^1^ Adjusted for age, sex, total energy intake and first-degree family history of colorectal cancer.

^2^ Adjusted for age, sex, total energy intake, first-degree family history of colorectal cancer, smoking, drinking, education, and obesity.
