# Supplementary material for: Interaction between vitamin E intake and a COMT gene variant on colorectal cancer risk among Korean adults: a case-control study
Source: Epidemiol Health. 2023 Nov 14;45:e2023100. doi: 10.4178/epih.e2023100 (PMC10876447; doi:10.4178/epih.e2023100)
Supplement: Supplementary file 2 [file epih-45-e2023100-Supplementary-2.docx]

| **Supplemental Material 2. Association between vitamin E density and colorectal cancer risk, stratified by *COMT* rs740603 genotype (recessive or codominant model)** | | | |
| --- | --- | --- | --- |
| ***COMT* SNP rs740603** | **Lower vitamin E^1^** | **Higher vitamin E^1^** | **P-value** |
| *Recessive* |  |  |  |
| G/G+A/G |  |  |  |
| Number of cases/controls | 374/323 | 265/316 |  |
| Model 1, OR (95% CI)^2^ | 1 (ref) | **0.64 (0.51, 0.81)** | **0.0002** |
| Model 2, OR (95% CI)^3^ | 1 (ref) | **0.72 (0.56, 0.92)** | **0.0075** |
| A/A |  |  |  |
| Number of cases/controls | 213/164 | 123/172 |  |
| Model 1, OR (95% CI)^2^ | 1 (ref) | **0.54 (0.39, 0.75)** | **0.0002** |
| Model 2, OR (95% CI)^3^ | 1 (ref) | **0.68 (0.48, 0.96)** | **0.0288** |
| *Codominant* |  |  |  |
| G/G |  |  |  |
| Number of cases/controls | 89/89 | 67/68 |  |
| Model 1, OR (95% CI)^2^ | 1 (ref) | 0.88 (0.54, 1.43) | 0.6109 |
| Model 2, OR (95% CI)^3^ | 1 (ref) | 1.13 (0.66, 1.91) | 0.6622 |
| A/G |  |  |  |
| Number of cases/controls | 285/234 | 198/248 |  |
| Model 1, OR (95% CI)^2^ | 1 (ref) | **0.58 (0.44, 0.76)** | **<0.0001** |
| Model 2, OR (95% CI)^3^ | 1 (ref) | **0.63 (0.47, 0.83)** | **0.0011** |
| A/A |  |  |  |
| Number of cases/controls | 231/164 | 123/172 |  |
| Model 1, OR (95% CI)^2^ | 1 (ref) | **0.54 (0.39, 0.75)** | **0.0002** |
| Model 2, OR (95% CI)^3^ | 1 (ref) | **0.68 (0.48, 0.96)** | **0.0288** |

^1^ Lower and higher vitamin E groups had vitamin E density at or below and above the median (5.03 mg/1,000 kcal), respectively.

^2^ Adjusted for age, sex, total energy intake and first-degree family history of colorectal cancer; p-for-interaction=0.3141 (recessive model); 0.1563 (codominant model).

^3^ Adjusted for age, sex, total energy intake, first-degree family history of colorectal cancer, smoking, drinking, education, and obesity; p-for-interaction=0.5827 (recessive model); 0.0661 (codominant model).
