# Supplementary material for: Interaction between vitamin E intake and a COMT gene variant on colorectal cancer risk among Korean adults: a case-control study
Source: Epidemiol Health. 2023 Nov 14;45:e2023100. doi: 10.4178/epih.e2023100 (PMC10876447; doi:10.4178/epih.e2023100)
Supplement: Supplementary file 3 [file epih-45-e2023100-Supplementary-3.docx]

| **Supplemental Material 3. Association between vitamins C and E density and colorectal cancer risk, stratified by *COMT* rs740603 genotype** | | | | |
| --- | --- | --- | --- | --- |
| ***COMT* SNP rs740603^1^** | **Lower vitamin E^2^** | | **Higher vitamin E^2^** | |
|  | **Lower vitamin C^3^** | **Higher vitamin C^3^** | **Lower vitamin C^3^** | **Higher vitamin C^3^** |
| G/G |  |  |  |  |
| Number of cases/controls | 66/66 | 23/23 | 15/15 | 52/53 |
| Model 1, OR (95% CI)^4^ | 1 (ref) | 0.74 (0.36, 1.52) | 0.82 (0.35, 1.93) | 0.81 (0.45, 1.43) |
| Model 2, OR (95% CI)^5^ | 1 (ref) | 0.67 (0.31, 1.46) | 0.94 (0.37, 2.36) | 1.03 (0.56, 1.91) |
| A/A+A/G |  |  |  |  |
| Number of cases/controls | 365/294 | 133/104 | 93/113 | 228/307 |
| Model 1, OR (95% CI)^4^ | 1 (ref) | 1.01 (0.74, 1.38) | **0.59 (0.43, 0.82)** | **0.55 (0.43, 0.70)** |
| Model 2, OR (95% CI)^5^ | 1 (ref) | 0.95 (0.68, 1.32) | **0.65 (0.46, 0.91)** | **0.61 (0.47, 0.79)** |

^1^ Dominant model

^2^ Lower and higher vitamin E groups had vitamin E density at or below and above the median (5.03 mg/1,000 kcal), respectively.

^3^ Lower and higher vitamin C groups had vitamin C density at or below and above the median (53.83 mg/1,000 kcal), respectively.

^4^ Adjusted for age, sex, total energy intake and first-degree family history of colorectal cancer; p-for-interaction between vitamins C and E=0.6259 (G/G); 0.7245 (A/A+A/G).

^5^ Adjusted for age, sex, total energy intake, first-degree family history of colorectal cancer, smoking, drinking, education, and obesity; p-for-interaction between vitamins C and E=0.4266 (G/G); 0.9993 (A/A+A/G).
